# The Reliability of the Wall Drop Punt Kick and Catch Test

**DOI:** 10.3390/jfmk8020072

**Published:** 2023-05-26

**Authors:** Rui Matos, Nuno Amaro, Nataniel Lopes, Pedro Costa, Miguel Jacinto, Filipe Rodrigues, Raul Antunes, Luís Coelho, Sergio J. Ibáñez, Diogo Monteiro

**Affiliations:** 1 ESECS—Polytechnic of Leiria, 2411-901 Leiria, Portugal; 2 Life Quality Research Centre (CIEQV), 2400-901 Leiria, Portugal; 3 Faculdad de Ciencia del Deporte, Universidad de Extremadura, 10003 Cáceres, Spain; 4 Center for Innovative Care and Health Technology (ciTechCare), 2415-396 Leiria, Portugal; 5 Research Center in Sport, Health, and Human Development (CIDESD), 5000-558 Vila Real, Portugal

**Keywords:** coordination, assessment, development, children, fundamental motor skills, object control

## Abstract

This study aimed to evaluate the reliability of a manipulative eye–segmental (hand and foot) coordination task, namely the Wall Drop Punt Kick and Catch test (WDPK&C), over two weeks. Forty-one children and adolescents (18 boys, 23 girls) with a mean age of 10.2 (SD = 1.62) years old were recruited for assessment. Subjects had 30 s to perform as many ball impacts as possible on a wall two meters away, following a drop punt kick, rebound on the wall, and catch sequence. The Intraclass Correlation Coefficient (ICC = 0.896) for unique measures, Cronbach Alpha (α = 0.945), and Lin’s Concordance Correlation Coefficient (CCC = 0.896) provide evidence of reliability considering two consecutive measurements. These results further support the reliability of the WDPK&C test in a sample of Portuguese children and adolescents. Thus, the WDPK&C test can be applied to Portuguese boy and girl children and adolescents. Forthcoming studies should test the reliability of this test across different age groups since it is intended to be a test with a wide lifespan coverage.

## 1. Introduction

Henderson and Sugden [1] define motor competence as a person’s ability to perform different motor actions, including coordinating fine and gross motor skills needed to handle daily tasks. Gallahue et al. [2] refer to it as the ability to be proficient in a broad range of locomotor, stability, and manipulative gross motor skills. Due to its close connection with and dependence upon coordination, having adequate coordination level is a solid step to become motor-competent. Research shows [3,4,5] that children with a good development of fundamental motor skills are more motor-competent and will more probably adhere to healthy physical activity habits compared to those with lesser motor competence. More specifically, manipulative gross motor skills (e.g., kicking, throwing, catching), even more than locomotor ones, are associated with greater participation in physical activity and the positive health-related consequences from activities that need manipulative gross motor skills [6,7].

Several motor batteries have been used to assess motor competence, such as the Test of Gross Motor Development (TGMD-3) [8], the Movement Assessment Battery for Children (MABC-2) [9] or the Körperkoordinationstest Für Kinder (KTK) [10]. The TGMD-3 is a standardized assessment tool used to measure the gross motor skills of children between the ages of 3 and 10. Similar to the original TGMD, the TGMD-3 assesses two broad categories of gross motor skills: locomotor skills and object control skills. In addition to the traditional skills assessed in the original TGMD, the TGMD-3 includes new skills such as leaping, horizontal jumping, and overhand throwing. The MABC-2 is a standardized assessment tool used to evaluate the motor skills and motor development of children between the ages of 4 and 16 years. The MABC-2 consists of three dimensions (with several subtests or tasks each): manual dexterity, aiming and catching, and balance. The manual dexterity evaluates fine motor coordination, using tasks like turning pegs. The aiming and catching evaluates gross motor skills, with tasks such as throwing a bean bag or catching a ball. The balance dimension assesses the child’s ability to maintain balance and control while standing on one foot, walking heel-to-toe, and performing other activities that challenge balance. Last, the KTK is a standardized assessment tool used to evaluate the motor coordination of children between the ages of 5 and 14 years. The KTK consists of four subtests: walking backwards, jumping sideways, hopping on one leg, and moving sideways. Each of the previous described test includes several tasks, and the child’s performance is scored based on specific criteria for each task. The scores are then combined to obtain a total score for the assessment. Recently, some researchers have proposed alternative or complementary tests to the gold standard test KTK (and KTK-3) such as the Motor Competence Assessment (MCA) [11], the Test of Motor Competence (TMC) [12] or the KTK-3+ Eye-Hand Coordination (KTK-3 + EHC) [13], either because the former do not allow for a lifespan vision or because they lack some important components, such as manipulative ones.

While these batteries provide a manipulative component, none incorporates a test where eye-hand and eye-foot coordination are jointly assessed. In other words, these assessments do not evaluate two motor components in the same task. Specifically, the KTK-3 + EHC developed by Platvoet et al. [13] uses Faber et al.’s [14] task to complete their proposed battery (i.e., KTK-3 + EHC). EHC requires the individual to throw a tennis ball to the wall with one hand and catch it with the other (for 30 s), but no eye–foot test is used. Sigmundsson et al. [12] TMC requires subjects to manipulate objects with hands but not with feet. Finally, Luz et al. [11] MCA uses manipulative components with hands and feet (kicking and throwing in distance), but they are performed in a separate way as two independent tests.

Matos et al. [15] created the Wall Drop Punt Kick and Catch (WDPK&C) test, which became a new gross manipulative coordination test. This motor skill is very interesting from the motor coordination point of view since it directly implies the manipulation of an object (ball) with the upper (dropping and catching) and lower (kicking) limbs and not just by one or another. This characteristic brings an increased complexity factor that may be adduced as an interesting contribution for the motor competence and coordination research line. Although the results are promising, the authors [15] stated that several intermediate steps still had to be performed before the WDPK&C could become a gross manipulative (eye–hand and eye–foot) coordination test and a potential candidate to integrate some of the referred to motor competence batteries. Thus, the reliability of the WDPK&C as a measure of gross motor development has not been established. One of those steps would be to determine its reliability (via reproducibility) over a period of one to two weeks for the WDPK&C in a sine qua non condition. In fact, Matos et al. [15], due to pandemic onset, could only calculate ICC and Cronbach’s alpha for the two trials on the same testing session. Although these were positive (ICC value of 0.94 and Cronbach’s alpha coefficient of 0.97), it is still necessary to perform the between-sessions reliability analysis.

Analyzing the reliability of the previously mentioned motor tests or batteries, it is possible to conclude that procedures and results from statistical tests seem not to be completely convergent, either on the interval between consecutive applications, the obtained Intraclass Correlation (ICC) coefficient or results interpretation. Sigmundsson et al. [12] reported a global ICC for their TMC battery of 0.87, ranging between 0.75 and 0.94 considering individual subtests in an adult sample, with measurements one week apart. Rebelo-Gonçalves et al. [16], analyzing the reproducibility of an eye-foot coordination test in adolescent futsal players, recorded the values of 0.79 and 0.72 using the same test with two different balls, with measurements one week apart. Hoeboer et al. [17] considered the Athletic Skills Track (AST) among 4- to 12-year-old children and reported ICC values that ranged from 0.80 to 0.88, considering three age-bands assessed and a test–retest procedure with a two-week interval. Silva et al. [18], with a sample of adolescent girl volleyball players, found ICC values that ranged from 0.72 to 0.99 on the MCA battery, although the test-retest procedure was only considered within a session with two or three trials and not between sessions. Finally, Smits-Engelsman et al. [19] studied test–retest reliability of the Performance and Fitness (PERF-FIT) test battery with 72 children (33 boys and 39 girls, 5–12 years old), with an interval period between session one and two that varied from a minimum of one to a maximum of two weeks. Authors reported that 11 of the 12 studied items (e.g., fundamental motor skills such as throwing, catching, or bouncing) had an ICC of 0.80 or more, with only one poor ICC (for the jumping item).

Considering the applicability of the WDPK&C as an upper and lower limb motor test, the present study aimed to determine the reproducibility of this test over a period of two weeks, stated on the COSMIN checklist as an appropriate time interval in the case of test–retest reliability [20]. It is hypothesized that WDPK&C would present acceptable reliability across time [15].

## 2. Materials and Methods

### 2.1. Design

Prior to conducting the study, a sample size calculation for Intraclass Correlation Coefficient (ICC) analysis was performed using the Arifin calculator [21] to determine the necessary sample size to achieve adequate statistical power. The minimum acceptable reliability was estimated to be 0.70, with a desired significance level of 0.05 and power of 0.95. The calculated minimum sample size was 19. Following previous assumptions [22] to account for potential dropouts and missing data (10%), a minimum of 22 participants would need to be recruited.

### 2.2. Participants

A non-probability sampling method was followed. Considering a convenience sample, forty-one children and adolescents (18 boys, 23 girls) with a mean age of 10.2 ± 1.62 years were recruited to participate in this study. All participants practiced athletics as amateur athletes and reported no motor or perceptual limitations. Sample characteristics are reported in Table 1.

The study was conducted in accordance with the Declaration of Helsinki for research involving human participants [23]. Before data collection, ethical approval for the study was obtained from the first author’s institution ethical commission. Parents or legal tutors were contacted after receiving a favorable response from the ethical commission to conduct the investigation. The nature, ethics, and data collection protocols of the project were presented during this meeting. Following this phase, the parents or legal tutors signed informed consent for their children and adolescents to participate voluntarily in this research.

### 2.3. Wall Drop Punt Kick and Catch Test

Following Matos et al.’s [15] protocol, subjects were required to perform as many ball impacts on a wall as possible in 30 s, following a drop punt kick movement pattern. Specifically, subjects should drop the ball they grabbed and kick it to the wall without a ground rebound moment. Only wall ball impacts with subsequent successful ball catching should be considered. A line marked on the ground two meters away from a wall should not be stepped upon or surpassed during the kick action so that an impact could be considered valid. Subjects could enter the 2 m zone if the ball was retained there. Each performer was entitled to five warming-up repetitions before two consecutive trials with 30 s recovery between assessments, so that the best performance could be registered. The test was performed with a size 4 football ball with a soft touch. The wall had a clean valid zone of 5 m wide, marked with vertical band stripes, and over 4 m high (the minimum required). Subjects performed the task individually before attending an athletics training session.

### 2.4. Statistical Analysis

Means and standard deviation were calculated for the WDPK&C test. The reliability analysis was conducted considering the ICC coefficient to measure the degree of dependence among individuals within a higher-level grouping. The Cronbach Alpha to demonstrate that tests that have been constructed for research projects are fit for purpose in terms of internal consistency reliability was also calculated. While there are no standard values for acceptable ICC scores in every domain [24], ICC values less than 0.50, between 0.50 and 0.75, and 0.75 and 0.90 are deemed to be considered as poor, moderate, and good reliability, respectively [25,26]. Considering assumptions by Hair et al. [25] and Landis and Koch [27], higher Cronbach Alpha values indicate higher levels of reliability when interpreting internal consistency and coefficients above 0.70 represent adequate scores of reliability. Additionally, Lin’s Concordance Correlation Coefficient (CCC) was also used as a complementary test for evaluation of WDPK&C’s reproducibility, as it measures how well bivariate pairs of observations conform relative to a gold standard or another set: in the present case, if pairs of results on moments 1 and 2 keep close to a 45-degree line, the line that would represent a perfect match between those two moments, with a CCC of 1. Thus, following Altman’s [28] recommendations suggesting that CCC should be interpreted close to other correlation coefficients such as Pearson’s, results < 0.2 are to be classified as poor and >0.8 as excellent. The analyses were conducted in IBM SPSS STATISTICS (v.26 for Windows, SPSS Inc., Chicago, IL, USA) [29].

Bivariate Pearson correlations were conducted considering age, height, weight, body mass index, sports experience, and WDPK&C test at baseline and after two weeks. The significance level for rejecting the null hypothesis was set at 5% for all tests. The analyses were also conducted in IBM SPSS STATISTICS (v.26 for Windows, SPSS Inc., Chicago, IL, USA).

## 3. Results

Sample characteristics of the participants are summarized in Table 1. Regarding years of athletics sports practice, 51.2% of the total sample had 1 or 2 years and 48,8% had 3 to 5 years of experience. Looking at sex, 55.6% of the boys had 1 or 2 years of athletics experience and 44.4% had 3 or 4 years. On the other hand, 47.8% of the girls had 1 or 2 years of athletics experience and 52.2% had 3 to 5 years of experience.

Descriptive statistics of the two measures of the WDPK&C test are displayed in Table 2. The ICC and the Cronbach Alpha showed acceptable scores of reliability and internal consistency, respectively. Lin’s CCC was 0.896, indicating as an excellent reproducibility result. These results further support the reliability of the WDPK&C test in a sample of Portuguese children and adolescents.

**Figure 1:**
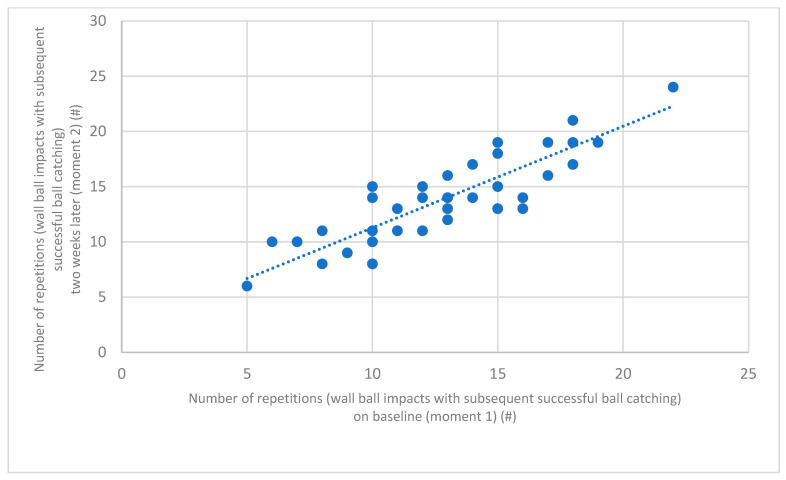
Individual results (*n* = 41) on WDPK&C at baseline (moment 1) and two weeks after.

Additionally, Figure 1 graphically represents the difference between the results of each of the 41 subjects in the initial and final performances. Twenty-three participants (56.1%) enhanced their performance from the first to the second moments, ten (24.4%) registered no alteration, and seven (17.1%) worsened. The average difference between the two moments was 1.68 (±1.37) repetitions (wall ball impacts with subsequent successful ball catching). Ten of the forty-one subjects (24.4%) achieved the same result on both moments and another ten (24.4%) altered their performance just by a repetition. Nine (22%) differentiated their performances by two repetitions, eight (19.5%) by three, three (7.3%) by four, and one (2.4%) by five repetitions, the biggest difference. It is observable that pairs of results (*X* vs. *Y* axes results) follow, generally, the 45-degree line that represents the ideal match between moments 1 and 2.

The results of the bivariate correlations are reported in Table 3. Several significant bivariate correlations were noted. Age, height, and weight were positively associated with WDPK&C test at baseline and after two weeks (*p* < 0.001). WDPK&C test at baseline and after two weeks were positively associated with each other (*p* < 0.001).

## 4. Discussion

The present study aimed to determine the reproducibility of this test over a period of two weeks. We hypothesize that this test would provide adequate scores of reliability, considering previous application in other studies. This task requires not just one but two linked, fundamental manipulative motor skills (drop punt kicking and catching).

The results of the ICC, Cronbach Alpha coefficient, and Lin’s CCC support the hypothesis that this test provides sufficient reliability over time. Comparing current results with existing research, the present ICC score (0.896) provides acceptable stability. Specifically, the ICC coefficients in this study are similar to those results reported by Sigmundsson et al. [12], with a global ICC of 0.87 for their TMC battery; Rebelo-Gonçalves et al. [16], who displayed reliability values of 0.79 and 0.72 on an eye-foot coordination test among young futsal players; or even Hoeboer et al. [17], who reported ICC values that ranged from 0.80 to 0.88 on a motor skill competence test among 4- to 12-year-old children. Furthermore, the present results are similar to those reported by Silva et al. [18], who found ICC values ranging between 0.72 and 0.99 on the six MCA tests in a sample of girls’ volleyball youth players. However, it is important to note here that the latter only used one intra-session procedure (two or three attempts) and not between sessions. Finally, the present reported ICC is in line with Smits-Engelsman et al. [19], given that 11 of their 12 studied items, grounded on fundamental motor skills, had an ICC of 0.80 or more. As previously stated, the length of time between the test–retest assessments in the referred studies was not always the same, and it may have been determinant for the ICC result. In fact, e.g., a big elapse of time between the two sessions may bring some learning effects, or even maturational ones, detracting from reliability results. In our study, the time that mediated the two assessments was of two weeks, and the best out of two trials in each session were used for reliability analysis. We must be conscious that reliability results may also be affected by other behavioral and circumstantial variables. Especially with youngsters, there will always exist performance variability, namely because of changes in motivation and familiarization with the tasks [19] or due to the large intra-individual variability in motor performance and development [30,31].

Age, height, and weight were positively and significantly associated with WDPK&C test at baseline and after two weeks. These results support previous works [13,15,32] suggesting that anthropometric features and chronological age are associated with this and with other gross manipulative coordination tasks. First, age is an important determinant in the development of manipulative coordination. As youngsters get older, their motor skills increase, allowing them to carry out more complicated manipulating tasks. Their nervous system develops, allowing for better coordination of the muscles, eyes, and brain. This better coordination can help them with tasks such as writing, sketching, and playing with objects [33,34]. Secondly, height and weight can also influence manipulative coordination skills. For example, larger hand size that may accompany increasing height and increased strength from greater body mass can contribute to greater grip when manipulating objects, which may be determinant on this task that requires to catch firmly the ball when it rebounds from the wall. Furthermore, as could be seen from the participants’ characterization, the percentage of children with overweight or obesity was very low. This may explain why there was not a (negative) correlation between performance on this task and weight or BMI. In fact, the literature often reveals a negative association between these anthropometric variables and motor coordination or motor competence [35,36,37] Here, as there were so few cases of unbalanced weight status, that did not occur, i.e., body weight was not a negative factor in the task performance.

The WDPK&C test at baseline and after two weeks were positively associated with each other (*p* < 0.001), also supported by Lin’s Concordance Correlation Coefficient (CCC) of 0.869. Thus, these results further support the reliability of this gross manipulative coordination task since ICC, internal consistency coefficient, and bivariate correlation coefficients were all above cutoff or statistically significant.

### Limitations, Strengths, and Agenda for Future Research

One limitation of the present study was that the sample comprised only athletic children and adolescents, which limits the degree to which our findings could be generalized. Thus, forthcoming studies should test the reliability of the WDPK&C in other children and adolescents with different characteristics and sports experience. Future studies could also test the applicability of this test with regard to sample characteristics such as practiced sports. Additionally, in order for this test to become a referenced assessment related to lifespan motor competence, future studies should test the reliability across age bands.

## 5. Conclusions

The WDPK&C, an eye–foot–hand coordination task, was revealed to be stable and thus reliable over a two-week period of time. Therefore, this constitutes an important step towards future validation of a gross manipulative segmental coordination task that, due to its unique feature, joining eye–hand with eye–foot coordination, may offer a valid alternative in the future to other manipulative tasks on motor competence and coordination batteries.

## Figures and Tables

**Table 1:** Sample characteristics.

Participants	Sports Experience (Years)	Height (m)	Weight (kg)	BMI (kg/m^2^)	BMI (%)	Weight Status
Total Sample (*n* = 41)	2.44 ± 1.21	1.40 ± 0.10	34.35 ± 7.75	17.29 ± 2.22	53.54 ± 26.00	9.8% OW; 2.4 Obese
Boys (*n* = 18)	2.33 ± 0.97	1.36 ± 0.09	32.36 ± 8.32	17.28 ± 2.62	52.86 ± 8.90	11.1% OW; 5.6 Obese
Girls (*n* = 23)	2.52 ± 1.38	1.43 ± 0.10	35.71 ± 7.17	17.30 ± 1.91	54.08 ± 24.15	8.7% OW

Notes: OW = overweight.

**Table 2:** Results of study endpoints and reliability coefficients.

Moment 1 (Repetitions)	Moment 2 (Repetitions)	ICC	Cronbach Alpha	CCC
12.88 ± 4.14	13.93 ± 4.25	0.896	0.945	0.869

**Table 3:** Bivariate correlations.

Variables	1	2	3	4	5	6	7
Age (years)	1						
Height (m)	0.88 **	1					
Weight (kg)	0.73 **	0.84 **	1				
Body Mass Index (kg/m^2^)	0.35 *	0.40 *	0.83 **	1			
Sports Experience (years)	0.54 **	0.51 **	0.32 *	0.02	1		
WDPK&C test at baseline (repetitions)	0.41 **	0.33 *	0.35 *	0.26	0.16	1	
WDPK&C test after two weeks (repetitions)	0.42 **	0.35 *	0.36 *	0.27	0.17	0.89 **	1

**Notes:** * *p*< 0.05; ** *p*< 0.01.

## Data Availability

The data presented in this study are available on request from the corresponding author.
